# Pathway Enrichment Analysis with Networks

**DOI:** 10.3390/genes8100246

**Published:** 2017-09-28

**Authors:** Lu Liu, Jinmao Wei, Jianhua Ruan

**Affiliations:** 1 College of Information Technology and Engineering, Marshall University, 1 John Marshall Dr, Huntington, WV 25755, USA; 2 College of Computer and Control Engineering, Nankai University, 94 Weijin Road, Tianjin 300071, China; weijm@nankai.edu.cn; 3 Department of Computer Science, The University of Texas at San Antonio, 1 Utsa Cir, San Antonio, TX 78249, USA

**Keywords:** pathway, protein–protein interaction network, enrichment analysis, gene sets, random walk with restart

## Abstract

Detecting associations between an input gene set and annotated gene sets (e.g., pathways) is an important problem in modern molecular biology. In this paper, we propose two algorithms, termed NetPEA and NetPEA’, for conducting network-based pathway enrichment analysis. Our algorithms consider not only shared genes but also gene–gene interactions. Both algorithms utilize a protein–protein interaction network and a random walk with a restart procedure to identify hidden relationships between an input gene set and pathways, but both use different randomization strategies to evaluate statistical significance and as a result emphasize different pathway properties. Compared to an over representation-based method, our algorithms can identify more statistically significant pathways. Compared to an existing network-based algorithm, EnrichNet, our algorithms have a higher sensitivity in revealing the true causal pathways while at the same time achieving a higher specificity. A literature review of selected results indicates that some of the novel pathways reported by our algorithms are biologically relevant and important. While the evaluations are performed only with KEGG pathways, we believe the algorithms can be valuable for general functional discovery from high-throughput experiments.

## 1. Introduction

Modern molecular biology has been revolutionized with the emergence of high-throughput experimental technologies such as microarrays and next-generation DNA sequencing. While many exciting discoveries have been made by data-driven analysis of such whole-genome data sets, an important problem that many biologists face everyday is how to interpret such large-scale data sets. A typical output from such a high-throughput experiment is a list of genes that are observed to be associated with a certain phenotype, such as those differentially expressed in tumors compared to normal tissues. In contrast to the easiness in obtaining the gene list, the bottleneck usually lies in understanding the meaning of the genes and generating new testable hypotheses with the hope to reveal the underlying molecular cause of the phenotype.

Biologists are knowledge-driven. A principled way to interpret such gene lists is to compare them with a database of well-annotated gene sets, such as biological pathways. For example, one of the most widely used approach, Over Representation Analysis (ORA) [1], counts the number of common genes shared by an input gene set and each annotated gene set and applies a statistical test, such as the cumulative hyper-geometric test, to calculate the statistical significance of the overlap. A *p*-value cutoff, e.g., 0.05, is then applied to select the annotated gene sets that share a statistically significant overlap with the input gene set. ORA is very easy to implement, and the idea behind it is straightforward to biologists. A popular extension of ORA, known as the Gene Set Enrichment Analysis (GSEA) [2], tries to eliminate the need for an ad hoc cutoff (e.g., expression fold change), which is often used in defining the input gene set. GSEA works by ranking all genes in the genome according to, say, level of differential expression, and tests whether any annotated gene set is ranked unexpectedly high or low through a running-sum statistic. While GSEA is becoming more popular, it is sensitive to noise and may report too many pathways that are conceptually hard to comprehend by biologists. In addition, GSEA is not applicable in cases where a completely ranked gene list is unavailable. As a result, ORA is still widely used by biologists.

Both ORA and GSEA depend on the availability of trusted gene annotations, such as gene ontology (GO) or metabolic pathways, which limits their applicability to only well annotated species. In addition, gene annotations in databases such as GO or Kyoto Encyclopedia of Genes and Genomes (KEGG) pathway may be strongly biased by some classes of genes or phenotypes that are popular targets, such as cancer. More importantly, it is becoming more and more well known that such enrichment-based analyses, including both ORA and GSEA, have very low discriminative power, as they treat genes as independent functional units. In reality, genes function in a highly coordinated way. For example, two gene sets may share few genes but can be involved in similar functional pathways, or they can represent two sub-modules of the same pathway. Common enrichment-based analysis may not be able to detect the relationship between gene sets.

To address the aforementioned issues, several studies have proposed the use of biological networks, such as protein–protein interaction (PPI) networks, as a more unbiased tool to investigate the biological meaning of gene sets [3,4,5,6]. The rationale is that genes that are located within a short distance in the same network are likely involved in similar biological processes. As such networks are typically obtained from high-throughput experiments, they are less likely to be biased by existing knowledge and can probably provide better coverage to different classes of genes and phenotypes. Furthermore, network-based analysis allows the relationship between genes to be explicitly modeled, instead of treating them as independent entities. While conceptually interesting, such methods have had limited success because high-throughput biological networks are usually very noisy, and still have many missing edges. Furthermore, the results obtained by such analyses, often in the form of PPI subnetworks, can be difficult to interpret because functional connections to biological processes are missing.

Another strategy, which seems to be more successful in practice, is to combine both biological networks and pathways in an analysis. For example, Alexeyenko et al. proposed a network-based method to investigate the associations between input gene sets and annotated gene sets by counting the number of network links between members of two gene sets [7]. Later, Glaab et al. proposed an algorithm called EnrichNet, which extends the method of [7] to include gene pairs that are not necessarily direct neighbors but are within close proximity in the network [8]. These approaches take gene correlations and interactions into consideration and agree with the fact that genes function in a coordinated way, which is a meaningful improvement over ORA. However, EnrichNet only provides scores to measure the functional associations and does not provide information about the statistical significance of the scores.

In this paper, we propose two network-based pathway enrichment analysis algorithms, NetPEA and NetPEA’, for conducting a network-based pathway enrichment analysis. Our algorithms consider not only shared genes but also gene–gene interactions. The two algorithms share some common features to identify hidden relationships between an input gene set and pathways, but each uses a different randomization strategy to evaluate statistical significance and, as a result, emphasize different pathway properties.

The remainder of this paper is organized as follows. We present the details of the two algorithms and the data sets in Section 2. In Section 3, we present the test results of our methods on multiple data sets and discuss the significance of our finding. We conclude with some remarks for future improvements in Section 4.

## 2. Methods and Materials

### 2.1. Overview of Our Algorithms

We propose two network-based pathway enrichment analysis algorithms, NetPEA and NetPEA’. NetPEA treats gene interactions as important as shared genes, while NetPEA’ is devised to find hidden causal pathways that are not enriched within the input gene set.

The two algorithms share their first step to calculate similarity scores between the input gene set and pathways based on their “closeness” on a PPI network, measured by a random walk with restart procedure. The two methods then adopt different randomization strategies as their background models to evaluate the statistical significance of the similarity scores. NetPEA uses randomized gene sets as its background; NetPEA’ employs randomized gene sets and randomized networks as its background. Both algorithms take gene interactions into account but focus on different pathways. Considering not only shared genes but also interacting genes, NetPEA extends the search scope of ORA, and therefore it reports more significant pathways. On the other hand, NetPEA’ attempts to de-emphasize pathways that are considered statistically significant simply because of their overlaps with the input gene set, and, as a result, it is able to identify pathways that are within close proximity to the input genes but do not have a significant overlap with them. As shown in Section 3, these hidden pathways, which are typically ignored by ORA and similar approaches, may very likely be the actual causal pathways and can be robust among experiments.

### 2.2. Random Walk-Based Similarity Measure

**Figure 1:**
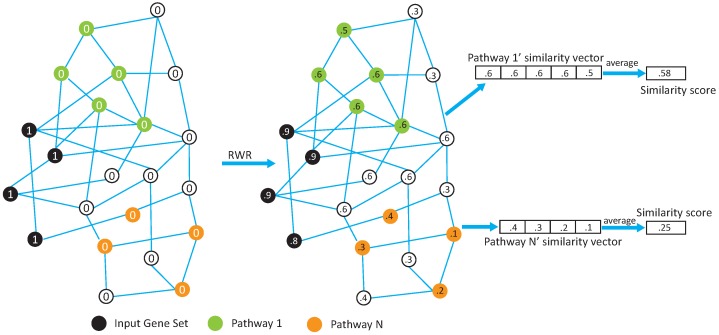
The workflow for calculating similarity scores between the input gene set and pathways. RWR refers to a Random Walk with Restart procedure.

Figure 1 shows the main component of our methods, calculating similarity scores, which is used to measure the closeness of pathways to the input gene set. First, we map the genes in the input gene set and pathways to a relevant biological network (PPI network in this study). Then all nodes are assigned an initial value of 0, except the ones in the input gene set which are assigned a value of 1. The Random Walk with Restart (RWR) procedure [9] is then used to spread the nonzero initial values to other nodes in the network. RWR is a well-known machine learning algorithm used to measure the similarities between nodes by imagining that, starting from each nonzero node, there is a random walker that, at each step, either moves to a randomly chosen neighbor or jumps back to the starting node. We formulate the procedure in Equation (1). *V* denotes the vector of initial node values; *p* represents the restart probability, which indicates the probability for random walkers to jump back to their starting nodes (fixed at 0.5 in this study); *M* is the PPI network transition matrix; and *S*_*n*_ is a vector of all nodes in the network, which is used to measure the similarities between each node in the network and nodes in the input gene set after *n* rounds of propagation. At the very beginning, *S*_0_ is initialized with *V*. After a period of time of propagation, *S*_*n*_ reaches some dynamic balance and the node values converge and become stable. For each pathway, we take the average of its member gene values as its similarity score to the input gene set.

(1) $$S_{n}=\left(1-p\right)*M*S_{n-1}+p*V$$

### 2.3. Network-Based Pathway Enrichment Analysis

To evaluate the statistical significance for pathways to achieve such similarity scores, we introduce two algorithms based on different randomization strategies.

#### 2.3.1. Algorithm NetPEA

NetPEA only randomizes the input gene set, in which we randomly choose the same number of genes as the input gene set to calculate the similarity score for each pathway. After we repeat this randomization 1000 times, we then get 1000 similarity scores for each pathway as its background. Equation (2) is used to calculate *z-scores* for pathway significance. *D* is the similarity score using gene set of interest as input, while *R* is a set 1000 similarity scores taking randomized gene sets as inputs.

(2) $$z-score=\frac{D-mean(R)}{std(R)}$$

We rank the pathways in descending order according to their *z-scores*. As the distribution of the *z-scores* roughly follows a normal distribution, we also convert the *z-scores* to *p*-values under a normal distribution assumption. We then select pathways with *z-scores* greater than 1.65, which corresponds to a *p*-value 0.05, as statistically significant pathways.

#### 2.3.2. Algorithm NetPEA’

NetPEA’ randomizes both the input gene set and PPI network to calculate the statistical significance of the associations between the input gene set and annotated gene sets. To randomize the network, we rewire the network connections randomly but ensure all nodes in the network maintain the same degrees as in the original PPI network. Repeating this rewiring 10 times, we have 11 networks including the original human PPI network. For each network, we perform a random walk with a restart procedure to calculate the similarity scores between the true input gene set and each of the annotated gene sets. This is also repeated for 1000 randomized input gene sets.

The *z-score* of the association between an input gene set and each annotated gene set is calculated by Equation (3). For each pathway, *DN* represents the similarity score taking the real input gene set and human PPI network as input; *DR* is a set of 10 similarity scores using the real input gene set and randomized networks as input; *RN* represents a set of 1000 similarity scores taking randomized gene sets and the human PPI network as input; and *RR* is 10 sets (each set corresponding to one randomized network) of 1000 similarity scores taking randomized gene sets and randomized networks as input. Similarly as in NetPEA, we rank all pathways according to their *z-scores*, and use a cutoff 1.65 to select statistically significant pathways.

(3) $$z-score=\frac{\left(DN-mean(DR))-mean(RN-mean(RR)\right)}{std(RN-mean(RR))}$$

Consider that there is a modest overlap between an input gene set and a particular pathway, and that none of the genes in the input gene set, other than those overlapping, are located within close proximity to the pathway genes in the PPI network. In this case, the expected values in the vector *DR* are close to *DN*. As a result, the *z-score* for this particular pathway will be “corrected” and becomes insignificant in NetPEA’ compared to that in NetPEA. Therefore, we expect NetPEA’ to report less pathways than NetPEA but, at the same time, be able to promote the ranking of the true causal pathways, which may not be ranked high in NetPEA or other overlap-based methods.

### 2.4. Data Sets

The annotated gene sets used in this study are from KEGG pathways [10]. We test our algorithms using input gene sets from several well-cited sources, including a Parkinson’s disease gene set [11], a lymphoma cancer gene set [12], two breast cancer gene sets obtained by two groups [13,14], two lung cancer gene sets [15,16], a diabetes disease gene set [17], a leukemia gene set [18], and two unpublished gene sets, “gender” and “p53” from the GSEA website [2]. All gene sets except “Parkinson” are from high throughput experiments. The Parkinson’s disease gene set is from a literature search. For four of the data sets, (p53, gender, diabetes, leukemia), genes are further categorized into “up” and “down” according to the direction its expression level changes in the experiments. The human PPI network is downloaded from the Human Protein Reference Database (HPRD, version 9) [19].

## 3. Results and Discussion

Validation of associations between genes sets is difficult because of the lack of ground truth and the biases inherent in different evaluation standards. To evaluate the performance of our methods and have a fair comparison with the existing ones, we adopted and designed multiple evaluation methods.

### 3.1. Validation Using KEGG Pathways as Input Genes

To validate that our algorithms can indeed identify the most relevant pathways, we first used KEGG pathways as input genes to identify the most significantly associated KEGG pathway for each pathway. The rationale is that each pathway should have a closer relationship with itself. Indeed, NetPEA ranks each pathway itself as the most enriched pathway with a very significant *z-score*. Moreover, some between-pathway associations found by NetPEA are also reasonable. For example, the top three pathways associated with “DNA replication” are “DNA replication”, ”mismatch repair”, and “nucleotide excision repair”, while the top three pathways associated with “chemokine signaling” are “hemokine signaling”, “cytokine–cytokine receptor interaction”, and “gap junction”.

On the other hand, NetPEA’ ranks the pathway itself as the most enriched pathway only for 62% pathways, and ranks the pathway itself in the top 10% for 91% of pathways. The deviation from the ground truth is because NetPEA’ is intended to explore hidden pathways by de-emphasizing pathways that are considered significant simply because of their overlaps with the input gene set. Indeed, for a number of cases where the input gene set itself is not ranked as the top gene set, we found evidence of the association between the reported top gene set and the input gene set, such as “maturity onset diabetes of the young” and “methane metabolism”. Note also that the association between the two pathways is not significant according to NetPEA.

### 3.2. Validation Using GSEA Outputs as Benchmarks

For four data sets that we have access to the coupled microarrays and ranked gene lists, we applied GSEA to rank the pathways and we use the rankings as benchmarks. GSEA is a benchmark widely used to validate gene set rankings, and [8] uses it to check pathway rankings. While the results may be biased, it provides partial evidence that our algorithms achieve better performances. Here we calculate Spearman correlation coefficients between each mentioned method and GSEA. Table 1 shows that for each input gene set, the largest correlation coefficient is from NetPEA or NetPEA’, which means that our algorithms gain more support on pathway rankings and are better than ORA and EnrichNet.

### 3.3. Evaluation Based on Number of Enriched Pathways

We apply our algorithms, NetPEA and NetPEA’, to each of the data sets mentioned in Section 2.4. Meanwhile, we run ORA on these input gene sets and compare the significant pathways found by the three methods.

#### 3.3.1. NetPEA vs. ORA

Table 2 shows significant pathways only reported by NetPEA but not present in the results for ORA. For most of cases (11/14), ORA does not identify any pathway that is not found by NetPEA. For common significant pathways discovered by both methods, we define NetPEA≪ORA, NetPEA≫ORA and NetPEA≈ORA by the ratios of their *p*-values. NetPEA≫ORA means the *p*-value ratio (NetPEA/ORA) is less than 0.001; NetPEA≪ORA represents the ratio greater than 1000; otherwise it is NetPEA≈ORA. Strikingly, no pathways fall into the range NetPEA≪ORA. Overall, NetPEA can successfully identify nearly all significant pathways reported by ORA. Moreover, NetPEA reports many significant pathways not found by ORA. The superiority of NetPEA over ORA can be explained by the fact that NetPEA not only considers the pathway enrichment caused by common genes but also takes gene interactions into account. Through the gene interactions, some pathways not enriched in ORA are elevated to be significant. In Section 3.6, we will show that these additional pathways are biologically meaningful.

#### 3.3.2. NetPEA’ vs. ORA

As NetPEA’ is devised to complement NetPEA, it provides new significant pathway information that is not present in the results of ORA or NetPEA. Compared to information in Table 2, the number of common significant pathways decreases for each input gene set (Table 3). At the same time, NetPEA’ produces some significant pathways not present in ORA. This difference is because of the network randomization in NetPEA’, which eliminates some significant pathways in ORA or NetPEA with loose gene interactions and lifts insignificant ones in ORA with close gene interactions. As shown clearly in Section 3.4, these pathways are often preserved between different experiments for the same disease, signifying the importance of the pathways for the disease.

### 3.4. Evaluation Using Cross-Data Stability Analysis

**Figure 2:**
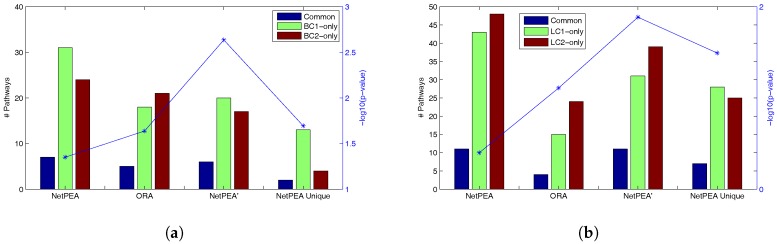
Common pathways from two different datasets of the same disease. (**a**) Overlap between two breast cancer data sets; (**b**) Overlap between two lung cancer data sets.

It is well known that the agreement is often poor between different high throughput experiments concerning the same disease performed by different groups. Among other reasons, this is because many of the genes identified by these experiments are caused by downstream effects, which can vary significantly among experiments. It is reasonable to assume that if indeed we can find true causal genes/pathways, agreement between experiments will be improved. Therefore, for the genes identified from the two breast cancer data sets and the two lung cancer data sets, we compare the significant pathways from different data sets reported by NetPEA, NetPEA’ and ORA. We calculate the ratios of common significant pathways to the total number of unique significant pathways identified from the two experiments as well as the *p*-values of the overlap under the hypergeometric distribution using 208 total KEGG pathways as background. Table 4 shows that NetPEA can find more common significant pathways than ORA and NetPEA’ as a result of the increased number of significant pathways reported by NetPEA, which partially suggest that the additional pathways reported by NetPEA are reasonable. Remarkably, while NetPEA’ reports much fewer pathways than NetPEA, the number of common pathways remains almost unchanged. As shown in Figure 2, the statistical significance of overlap between the pathways detected from different data sets is the highest in NetPEA’, compared to NetPEA and ORA. Therefore, we believe that the pathways reported by NetPEA’ may have a greater chance of containing the pathways that are directly associated with the phenotype instead of downstream effects. In addition, while the overlap of pathways detected by NetPEA is not as significant as in ORA, this is mainly due to the increased number of detected pathways and the limited number of candidate pathways as background. Since almost all pathways reported by ORA are also reported by NetPEA, we removed the ORA-detected pathways from NetPEA results and reanalyzed the overlap. As shown in Figure 2 (“NetPEA unique”), the pathways found by NetPEA but not by ORA do have an increased level of overlap compared to ORA, suggesting that the additional pathways identified by NetPEA are biologically relevant. Collectively, the results suggest that our algorithms have an advantage in interpreting the results of high throughput experiments performed by different groups and can potentially discover the key pathways underlying the diseases.

### 3.5. Pathways Cross Verification Analysis

Checking whether pathways ranked at the top by one method are also ranked at the top by other methods can provide additional confidence to biologists and help biologists to narrow down new hypotheses to test. Here we use two cross verification methods, positive cross verification and negative cross verification, to compare our algorithms with ORA, EnrichNet and GSEA. For positive cross verification, we examine how many pathways out of the top 20 by one method appear in the top 20 pathways determined by all the other methods. For negative cross verification, we checked how many pathways out of the top 20 by one method are ranked below the top 100 by the other three methods. We verify NetPEA and NetPEA’ separately because if we verify them together they may vote for each other, which would provide biased, favorable results for our algorithms.

#### 3.5.1. NetPEA

Table 5 shows that ORA receives the most recognition and reports only one pathway that is not agreed by others, which is understandable because ORA is the most conservative method and most of its results are also reported by NetPEA and EnrichNet. For the two network-based approaches, they receive similar results on positive cross verification, while NetPEA is better than the counterpart with less negative results. Moreover, the only pathway of negative verification result of NetPEA is “taste transduction” for diabetes, which has been reported previously [20]. The pathways of EnrichNet’s negative verification result include “thyroid cancer”, “basal cell carcinoma”, “melanogenesis”, “endometrial cancer” and “hedgehog signaling”. Our limited literature search does not reveal enough evidence of their associations with diabetes. GSEA is the one receiving the least recognition as it exploits whole microarrays and, from a methodology point of view, it is far away from the other three methods. Its negative cross verification results include “olfactory transduction”, “mismatch repair” and “snare interactions in vesicular transport” for diabetes. These associations claimed by GSEA are hard to understand. Therefore, NetPEA has an advantage over other methods to rank meaningful pathways at the top.

#### 3.5.2. NetPEA’

Compared with Table 5, Table 6 shows that NetPEA’ receives less support than NetPEA. This is reasonable since NetPEA’ eliminates some pathways that are an important part of ORA. On the other hand, we conclude that NetPEA’ shares some similarities with GSEA because the results of positive verification of GSEA increase while its negative verification results decrease.

### 3.6. Novel Pathways

As shown in Section 3.3, our algorithms usually report more significant pathways than ORA. A careful inspection of these additional pathways suggests that many of them are biologically relevant and important. Here we only discuss a few of these pathways.

For the diabetes down-regulated input gene set, NetPEA ranks the pathway “glycerolipid metabolism”, as 5th with a significant *z-score* 3.5 (*p*-value = $2.3\times 10^{-4}$), and NetPEA’ ranks it as 1st with a significant *z-score* 7.9 (*p*-value = $1.4\times 10^{-15}$). The same pathway has a *p*-value 0.12 in ORA and is ranked 37th by EnrichNet. Extensive literature review shows that “glycerolipid metabolism” plays an important role in the pathogenesis of obesity and type 2 diabetes [21,22].

Another good example is the Leukemia up-regulated input gene set, where NetPEA’ ranks the pathway “chronic myeloid leukemia” 4th with a significant *z-score* 3.12 (*p*-value = $9.0\times 10^{-14}$). ORA ranks it 63rd with an insignificant p-value, and EnrichNet ranks it 77th. This pathway is missed by NetPEA (*z-score* = 0.23). For the Leukemia down-regulated gene set, both NetPEA and NetPEA’ rank “folate biosynthesis” as the most significant pathway (*z-score* = 5.0 and 6.5 respectively), while the same pathway is ranked 22nd in EnrichNet and has a *p*-value 0.05 in ORA. A search through the literature confirms that the relationship between the pathway, “folate biosynthesis”, and leukemia can be verified [23].

Other verifiable significant associations that are identified by our methods but missed by both EnrichNet (rank > 30) and ORA (*p*-value > 0.05) include “pathways in cancer” for the p53 up-regulated gene set, “steroid hormone biosynthesis” and “sphingolipid metabolism” in Parkinson’s disease, “natural killer cell mediated cytotoxicity” in diabetes, as well as “MAPK signaling”, “ERBB signaling”, “PPAR signaling”, “focal adhesion” and “ECM receptor interaction” for various cancer gene sets, to mention a few.

## 4. Conclusions

In this paper, we propose two novel network-based algorithms to analyze functional associations between input gene sets and annotated gene sets (e.g., KEGG pathways). The two algorithms apply different randomization strategies to evaluate the statistical significance of the associations and often return complementary results. Compared to the well-adopted over representation analysis (ORA), our methods extend beyond overlap-based comparison, and as a result they are able to identify more significant pathways, report more common pathways shared by different gene sets of the same diseases and gain more GSEA support on the pathways rankings. Compared to another network-based approach, EnrichNet, our algorithms usually report fewer false negative pathways, have a better discriminative power and provide statistical significance. We demonstrate that novel significant pathways reported by our algorithms are biologically meaningful and are confirmed by previous publications. In the future, we plan to extend the methods to be applied to multiple heterogeneous terms and contexts.

## Figures and Tables

**Table 1:** Spearman correlation coefficient between GSEA and four other approaches.

**Input Gene Set**	**p53 (down)**	**Gender (down)**	**Diabetes (down)**	**Leukemia (down)**
NetPEA	**0.4653**	**0.2713**	0.2373	**0.3253**
NetPEA’	0.3978	0.1265	**0.2401**	0.182
ORA	0.3968	0.2406	0.1602	0.264
EnrichNet	0.2967	0.219	0.1779	0.2726
**Input gene set**	**p53 (up)**	**Gender (up)**	**Diabetes (up)**	**Leukemia (up)**
NetPEA	**0.3427**	**0.4349**	**0.227**	0.1195
NetPEA’	0.2911	0.2756	0.1419	**0.1438**
ORA	0.2507	0.332	0.1421	0.0583
EnrichNet	0.2167	0.3067	0.0823	0.0599

**Table 2:** Significant pathways: NetPEA vs. ORA .

Input Gene Set	# Unique Pathways		# Common Pathways		
	NetPEA	ORA	PNetPEA≫PORA	PNetPEA≪PORA	PNetPEA≈PORA
Parkinson	**18**	0	19	0	18
Lymphoma	**18**	0	10	0	5
Breast cancer [13]	**13**	0	6	0	12
Breast cancer [14]	4	1	4	0	16
Lung cancer [15]	**28**	0	3	0	12
Lung cancer [16]	**25**	1	6	0	17
Diabetes (down)	**13**	0	4	0	2
Diabetes (up)	**7**	0	0	0	2
Leukemia (down)	**22**	0	1	0	2
Leukemia (up)	**7**	0	3	0	7
Gender (down)	**7**	0	0	0	1
Gender (up)	**10**	0	1	0	2
p53 (down)	**14**	0	1	0	5
p53 (up)	**24**	1	5	0	18

**Table 3:** Significant pathways: NetPEA’ vs. ORA.

Input Gene Set	# Unique Pathways		# Common Pathways		
	NetPEA’	ORA	$P{\mathrm{NetPEA}}^{\prime }\gg P\mathrm{ORA}$	$P{\mathrm{NetPEA}}^{\prime }\ll P\mathrm{ORA}$	$P{\mathrm{NetPEA}}^{\prime }\approx P\mathrm{ORA}$
Parkinson	7	28	3	1	5
Lymphoma	16	5	4	0	6
Breast cancer [13]	11	9	0	0	9
Breast cancer [14]	5	9	1	0	11
Lung cancer [15]	28	12	1	0	2
Lung cancer [16]	27	12	1	0	11
Diabetes (down)	19	4	0	0	2
Diabetes (up)	13	2	0	0	0
Leukemia (down)	16	2	0	0	1
Leukemia (up)	15	6	0	0	4
Gender (down)	5	1	0	0	0
Gender (up)	20	3	0	0	0
p53 (down)	13	5	0	0	1
p53 (up)	27	15	0	0	9

**Table 4:** Common significant pathways analysis.

	NetPEA	NetPEA’	ORA
Common pathways between two breast cancer data sets ([13,14])	glycolysis/gluconeogenesis, homologous recombination, oocyte meiosis, p53 signaling, progesterone-mediated oocyte maturation, base excision repaire, cell cycle	lipoic acid metabolism, progesterone-mediated oocyte maturation, cell cycle, protesome, ubiquitin mediated proteolysis, oocyte meiosis	glycolysis/gluconeogensis, homologous recombination, progesterone-mediated oocyte maturation, cell cycle, oocyte meiosis
Common pathways between two lung cancer data sets ([15,16])	DNA replication, ECM -receptor interaction, focal adhesion, mismatch repair, nucleotide excision repair, pancreatic cancer, pathways in cancer, prostate cancer, small cell lung cancer, base excision repair, bladder cancer	antigen processing and presentation, base excision repair, DNA replication, ErBB signaling, FC epsilon RI signaling, FC gamma r-mediated phagocytosis, lysosome, mismatch repair, nucleotide excision repair, prostate cancer, vibrio cholerae infection	focal adhesion, mismatch repair, pathways in cancer, small cell lung cancer

**Table 5:** Pathways cross verification analysis for NetPEA.

Input Gene Set	Positive				Negative			
	NetPEA	ORA	EnrichNet	GSEA	NetPEA	ORA	EnrichNet	GSEA
Lung cancer [15]	16	16	16	7	0	0	0	2
Lung cancer [16]	14	16	16	4	**0**	0	**1**	8
Diabetes (down)	17	19	16	5	0	0	0	3
Diabetes (up)	15	16	13	5	**1**	1	**5**	3
Leukemia (down)	18	18	15	5	0	0	0	7
Leukemia (up)	15	19	15	8	**0**	0	**1**	4
Gender (down)	18	19	15	8	**0**	0	**1**	2
Gender (up)	17	19	15	7	0	0	0	1
p53 (down)	19	19	13	10	**0**	0	**1**	2
p53 (up)	16	15	16	6	**0**	0	**2**	3

**Table 6:** Pathways cross verification analysis for NetPEA’.

Input Gene Set	Positive				Negative			
	NetPEA’	ORA	EnrichNet	GSEA	NetPEA’	ORA	EnrichNet	GSEA
Lung cancer [15]	7	14	16	9	0	0	0	2
Lung cancer [16]	4	15	15	4	4	0	1	7
Diabetes (down)	6	19	16	6	4	0	0	2
Diabetes (up)	8	15	13	5	1	1	4	2
Leukemia (down)	7	15	16	5	1	0	0	6
Leukemia (up)	7	18	15	9	1	0	0	2
Gender (down)	8	17	15	9	2	0	1	2
Gender (up)	9	18	15	10	2	0	0	1
p53 (down)	6	14	14	11	1	0	1	3
p53 (up)	6	13	15	8	0	0	2	2

## Abbreviations

The following abbreviations are used in this manuscript:

ORA	Over Representation Analysis
GSEA	Gene Set Enrichment Analysis
GO	Gene Ontology
PPI	Protein–Protein Interaction
RWR	Random Walk with Restart
